# Correction: Shawky et al. The Biological Impacts of Sitagliptin on the Pancreas of a Rat Model of Type 2 Diabetes Mellitus: Drug Interactions with Metformin. *Biology* 2020, *9*, 6

**DOI:** 10.3390/biology13070527

**Published:** 2024-07-16

**Authors:** Lamiaa M. Shawky, Ahmed A. Morsi, Eman El Bana, Safaa Masoud Hanafy

**Affiliations:** 1 Department of Histology and Cell Biology, Benha Faculty of Medicine, Benha University, Benha 13511, Egypt; lamiashawky1974@gmail.com; 2 Department of Histology and Cell Biology, Faculty of Medicine, Fayoum University, Fayoum 63511, Egypt; 3 Department of Anatomy, Benha Faculty of Medicine, Benha University, Benha 13511, Egypt; emanelbana88@gmail.com; 4 Department of Anatomy, Faculty of Medicine for Girls, Al-Azhar University, Cairo 11865, Egypt; safaahanafy4577@gmail.com

## Error in Figure

In the original publication [1], there was a mistake in Figure 7 as published. Image D was mistakenly selected for the metformin-treated group. The corrected Figure 7 and the updated magnification appears below. The authors state that the scientific conclusions are unaffected. This correction was approved by the Academic Editor. The original publication has also been updated.


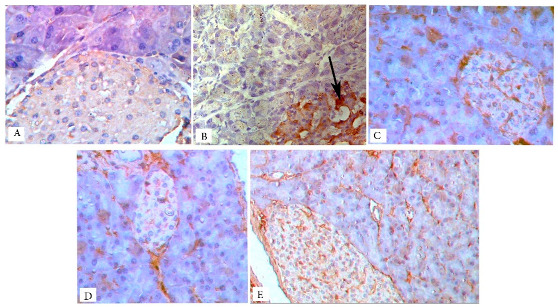


Magnification: (**A**–**D**) ×400 and (**E**) ×200

## References

[1] Shawky L.M., Morsi A.A., El Bana E., Hanafy S.M. (2020). The Biological Impacts of Sitagliptin on the Pancreas of a Rat Model of Type 2 Diabetes Mellitus: Drug Interactions with Metformin. Biology.

